# Effects of mangrove removal on benthic organisms in the Siangshan Wetland in Hsinchu, Taiwan

**DOI:** 10.7717/peerj.5670

**Published:** 2018-10-04

**Authors:** Yu-Chi Chen, Ta-Jen Chu, Ju-Der Wei, Chun-Han Shih

**Affiliations:** 1 Department of Landscape Architecture, Chung-Hua University, Hsin Chu, Taiwan; 2 Department of Tourism and Leisure, Chung Hua University, Hsin Chu, Taiwan; 3 Department of Civil Engineering, Chung-Hua University, Hsin Chu, Taiwan; 4 Department of Leisure Management, Tungnan University, New Taipei City, Taiwan; 5 Ocean College, Minjiang University, Fuzhou, China

**Keywords:** Siangshan Wetland, Mangrove removal, Habitat rehabilitation, Remote sensing

## Abstract

Mangrove degradation is a well-documented trend, but the spread of mangroves within the Siangshan Wetland in Hsinchu, Taiwan, runs contrary to that trend. The spread of mangroves changes the structure and functions of habitats for benthic organisms, causes infilling of estuaries and flooding and creates breeding grounds for small black mosquitoes. A large-scale mangrove-removal project was undertaken by the Hsinchu City Government from October 2015 to March 2016. They also investigated the consequences of mangrove removal on benthic organisms and adjacent habitats from October 2015 to September 2016, and the density, species count, Shannon–Wiener index (*H*′), and Pielou’s evenness index (*J*′) of the mangrove and non-mangrove regions were compared. In this study, we used satellite telemetry images to monitor fluctuations in mangrove density from 2006 to 2016. The non-mangrove region exhibited more variations than the mangrove region. After mangrove removal, species returned to their original habitats and noteworthy biological values significantly increased in the mangrove regions. This study presents evidence to argue that mangrove removal benefits benthic organisms. The results indicate that mangrove removal can be an appropriate habitat rehabilitation strategy for benthic organisms. The ecological findings of this study can inform coastal managers or other officials who seek to steward mangrove biomass.

## Introduction

Special wetlands along Taiwan’s coasts contain extremely rich biological and landscape resources. Hard and strong coastal engineering methods have often been used to protect the rapidly developing coastal zones (Chu et al., 2007). However, these biological habitats are sensitive and fragile; after a coastal environment has been destroyed, it can be restored or rehabilitated only with great difficulty. At present, more than 50% of Taiwan’s coast is artificially constructed (Shih et al., 2011a; Shih et al., 2011b). Generally, scholars think that natural coast habitats provide healthier ecosystems than those that are artificially constructed. Thus, the question of whether natural coast ecological habitats are preferable to artificially constructed coasts is important in coastal management.

Mangroves areas produce and support a multitude of land- and water-dwelling organisms (Nagelkerken et al., 2008). Fish use mangrove areas as crucial breeding habitats and nursery areas (Denis et al., 2016). Mangroves are beneficial for aquaculture and agriculture; mangroves can provide firewood and building material, medicines, and for other local subsistence items (Alongi, 2002; Walters et al., 2008). In coastal areas, mangroves provide numerous benefits, such as protecting communities along the coastal against natural disasters and hazards, for example, cyclones, tsunamis, and shoreline erosion (Saenger, 2002; FAO, 2007). Few studies have investigated the negative biological effects of mangroves. Several studies have shown that species abundance and biodiversity decline in mudflats when mangrove forests expand and invade the surrounding habitats (Lee & Shih, 2004; Lee & Yeh, 2009; Shih et al., 2011a; Shih et al., 2011b; Yang et al., 2013); mangroves reduce the atmospheric concentration of CO_2_ (DelVecchia et al., 2014). In subtropical estuarine wetlands, fishes, crabs, gastropods, prawns, and other megafauna require mudflats to serve as critical habitats. Mangrove removal, reconstruction of tidal creeks, and other rehabilitation projects can improve the diversity of habitats for mangrove organisms (Huang et al., 2010; Shih et al., 2015). To maintain tidal mudflats, mangrove seedlings were removed from the Hong Kong Mai Po Ramsar Site; the result was elevated biodiversity (WWF Hong Kong, 2006). In northern New Zealand, the Northland Regional Council granted an environmental permit (CON20031099401) to remove a 0.26-ha fringe of mangrove trees from Mangawhai Harbour to improve water access; this project was remarkable in that it enabled researchers to observe what ecological consequences removing mangroves has on estuarine ecosystems (Alfaro, 2010).

Satellite imagery has informed several studies (Ramirez-Garcia, Lopez-Blanco & Ocana, 1998; Murray et al., 2003; Matsushita et al., 2007; Lee & Yeh, 2009; Shih et al., 2011a; Shih et al., 2011b; George et al., 2018) that have examined the reach of wetland vegetation and have assessed the relationship between mangrove distribution and coastal changes (Allison & Lee, 2004; Fromard et al., 2004; Nakamura et al., 2004; Shih et al., 2011a; Shih et al., 2011b; Lonard et al., 2017).

Siangshan Wetland, located in Hsinchu, Taiwan, is an important muddy wetland with abundant species and biodiversity. In the 1980s, mangrove planting projects for coastal protection resulted in the unexpected spread of mangroves. The invading mangroves changed the structure and functions of the habitat for benthic organisms and caused infilling of estuaries, flooding, and invasions of the small black mosquito (*Forcipomyia taiwana*). Therefore, the Municipal Government of Hsinchu has launched some small-scale mangrove-removal projects since 2000; however, the speed of removal is slower than the expansion. A large-scale removal project was planned from October 2015 to March 2016.

Some studies have analyzed the effects of mangrove removal, but few have evaluated the effects of large-scale removal efforts. In this study, we used satellite telemetry and biological investigation to monitor the situation before and after mangrove removal. The results showed that mangrove removal might be a feasible approach for coastal management and ecological restoration. The study provide ecological records that serve as a reference for future mangrove deforestations in other areas and is an important case study in coastal management.

**Figure 1:**
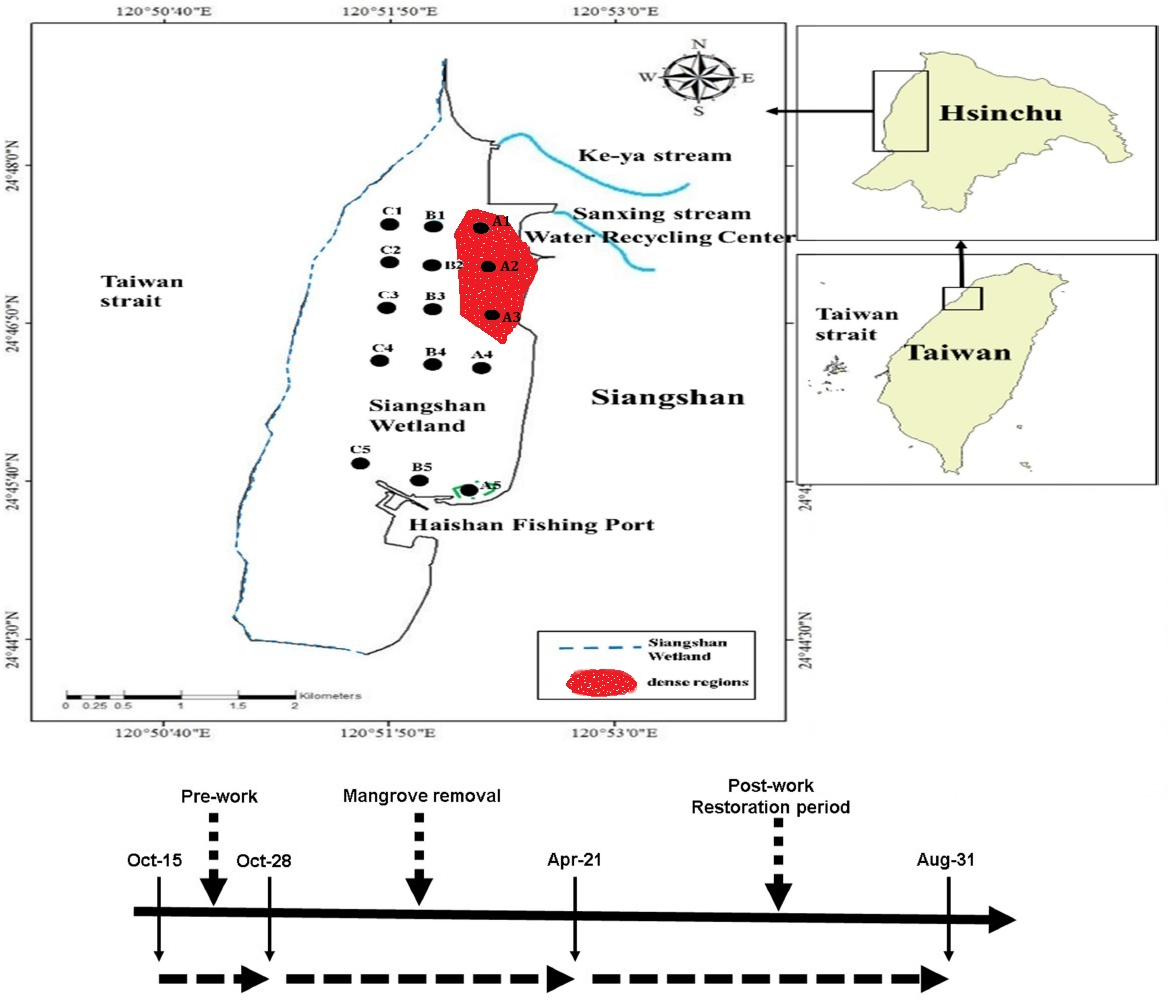
Sampling stations of macrobenthos.

## Materials and Methods

### Study area

**Figure 2:**
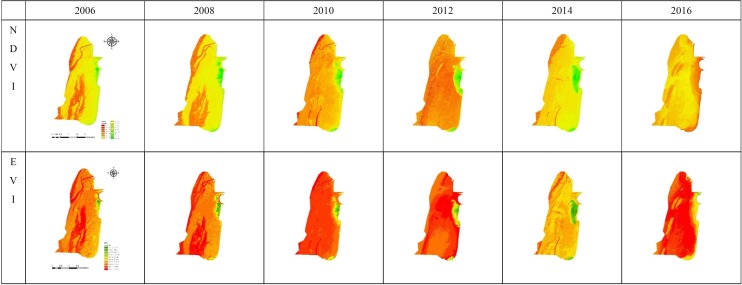
Image classification derived from NDVI and EVI.

The area studied in this work extends from the Sanxing stream to the Haishan Fishing Port, all of which lie to the west of Hsinchu, Taiwan. The coastline is approximately 8 km and the total study area occupies approximately 1,600 ha. This area is also known as the Siangshan Wetland (Fig. 1). In 2001, Siangshan Wetland was officially named the Hsinchu City Coastal Wildlife Sanctuary. The muddy intertidal zone is a breeding ground for large numbers of shrimp, crabs, shellfish, and benthic organisms, and it attracts a variety of protected bird species (National Important Wetland Conservation Project, 2014). The southwest portion of the wetland has large-scale oyster farms. The study area includes two dense mangrove regions and a smaller seedling area, as illustrated in Fig. 2. The northern dense region is located in the estuary of the Sanxing stream, and the other is in the northern part of the Haishan Fishing Port. The small seedling region is located between the two dense regions. The dominant mangrove species on the tidal flats are *Kandelia candel, Avicennia marina*, and *Rhizophora mucronata*. The crab population can reach very high densities and includes *Uca arcuata, Uca lacteal, Mictyris brevidactylus,* and *Macrophthalmus banzai*.

### Mangrove removal

The mangroves of Siangshan Wetland were planted in 1969, and a survey of mangrove stands in 1992 found as many as 5,300 stands. Mangrove areas were estimated in 0.1-ha areas by the Taiwan Endemic Species Research Institute in 1995. In 2000, the mangrove area covered approximately 107 ha, as reported by the Siangshan Wetland Mangrove Removal and Benefit Assessment Program of the Hsinchu Municipal Government. Because of the continuous spreading of the mangrove in the coastal areas, the effects seen included habitat singularity, decline of species abundance, decline of biodiversity, infilling of estuaries, flooding, and small black mosquito breeding. Several small-scale mangrove-removal projects, ranging from 1 to 14 ha, were implemented from 2007 to 2014. Related projects are all entrusted to NGOs.

Per the 2011 National Important Wetland Ecological Environment Investigation and Rehabilitation Project—Hsinchu City Wild Animal Sanctuary Habitat Rehabilitation Program guidelines, mangrove trees were cut with chain saws, manually transported to the shore, and transported by truck to incinerators.

To solve the problem of mangrove overspreading, a large-scale removal project was planned by the Hsinchu Municipal Government in October 2015. In this project, the mangrove forest was divided into two dense regions and a scattered region; a mechanical removal method was applied in the dense regions and manual removal in the scattered region. The process of mechanical removal is divided into five steps: trenching, shoveling, digging, compacting, and healing.

In the scattered region, workers used hoes to remove the branches and roots and then carried the debris to the shore for stacking. Finally, the waste was transported to incinerators in dump trucks. In March 2016, the cumulative removed mangrove area was 348 ha, which included 48 ha from the dense regions and approximately 300 ha from the scattered region (Table 1).

**Table 1:** The Hsinchu Municipal Government has launched some difference scale mangrove removal projects from 2006–2016.

Project year	Project number/Responsible unit	Removal method	Remove the area of mangroves (Hectares)
2007	SH096a1/The Society of Wilderness	Artificial	1
2008	SH097a1/The Society of Wilderness	Artificial /machine	3
2009	SH098a1/The Society of Wilderness	Artificial	5
2010	SH096/The Society of Wilderness	Artificial	14
2011	SH100a1/The Society of Wilderness	Artificial	5
2012	SH101a1/The Society of Wilderness	Artificial	5
2012	SH101a1/The Society of Wilderness	Artificial	4
2013	SH102a1/The Society of Wilderness	Artificial	3
2014	SH103a1/The Society of Wilderness	Artificial	5
2015	SH104a1/ EPA 102-078A /Taiwan Wetland Society	Artificial /machine	348

### Biological survey

Sampling for macrobenthos was conducted monthly from October 2015 to September 2016. The A, B, and C survey transects were set from north to south, and five sampling stations were positioned along each transect, forming a total of 15 sampling stations as shown in Fig. 1. The sampling stations A1, A2, A3, and A5 were located in the dense mangrove regions. Field experiments were approved by the Environmental Protection Administration, R.O.C. (Taiwan). The relevant permit approval number was ID: EPA 102-078A.

The Siangshan Wetland has semidiurnal and diurnal tides, and its tidal range is 2.7 m. Samples were collected on the tidal flats, and each sample was taken by excavating a frame (surface area, 1 m^2^) to a depth of 30 cm. Ten random frames were collected at each site for species identification, quantity calculation, and comparison before and after mangrove removal. The samples were immediately sieved (1 mm mesh size) and preserved in an 8% formaldehyde-seawater solution. The organisms were sorted and washed in a laboratory; species were identified, counted, and preserved in a 70% alcohol solution. Density was estimated as the count of individual organisms (N) per unit area; the total number of species (S) was applied to quantify species richness. The Shannon–Wiener index was applied to evaluate species diversity (*H*′, log) (Shannon & Weaver, 1963), whereas evenness (*J*′) was estimated by the method of Pielou (1966).

### Satellite telemetry survey

Multitemporal FORMOSAT-2 satellite images were analyzed to identify mangrove vegetation areas and nonvegetation areas. Satellite images (resolution, 4 m) were utilized to determine mangrove cover and its changes over time. Various satellite images taken at different times were compared to consider the spatial variation of mangrove vegetation; the images were separately collected on July 20, 2006, July 21, 2008, August 15, 2010, July 4, 2012, August 2, 2014, and July 17, 2016.

The Normalized Difference Vegetation Index (NDVI) and Enhanced Vegetation Index (EVI) are appropriate for vegetation studies (Ren et al., 2008; Son et al., 2013), and demonstrate a good range and sensitivity for monitoring and assessing spatial and temporal variations in vegetation amount and condition (Son et al., 2013). In this study, images were classified in two stages. First, the vegetation and nonvegetation areas were extracted from all satellite images by NDVI. Second, from within the extracted area, a minimum distance classification program then identified the mangrove and non-mangrove areas. Samples of mangrove areas and other vegetation areas from the digital aerial imagery served as training areas; mangrove boundaries were marked on the images. The types of mangroves, bare land, and water were analyzed using Erdas Imagine Software.

To calculate the area of mangrove cover, NDVI and EVI were used. Because plants highly reflect and absorb near-infrared and red wavelengths of light, the ratio of these two bands is often applied for vegetation mapping. A high ratio indicates thriving vegetation, whereas a low ratio suggests either stressed vegetation or the absence of vegetation. NDVI represents reaction to photosynthetic activity; the relevant equation is NDVI = (NIR − RED)/(NIR + RED) (Schowengerdt, 1997; Kovacs et al., 2004; Matsushita et al., 2007), where NIR represents the reflectance of near-infrared radiation and RED represents the reflection of visible red radiation, as measured by a satellite radiometer. NDVI can range from −1 to 1, with higher NDVI indicating larger vegetation cover (Lee & Shih, 2004; Lin, Lin & Chou, 2006; Matsushita et al., 2007; Lee & Yeh, 2009). The relevant equation is EVI = G × (*NIR* − *RED*)/(*NIR* + *C*1 × RED − C 2 × Blue + L) (Huete, Justice & Van Leewen, 1999), where EVI is a modified NDVI with enhanced vegetation monitoring ability, achieved by decoupling the cover background signals and the atmospheric interference (Huete, Justice & Van Leewen, 1999).

## Results

### Satellite telemetry results

**Figure 3:**
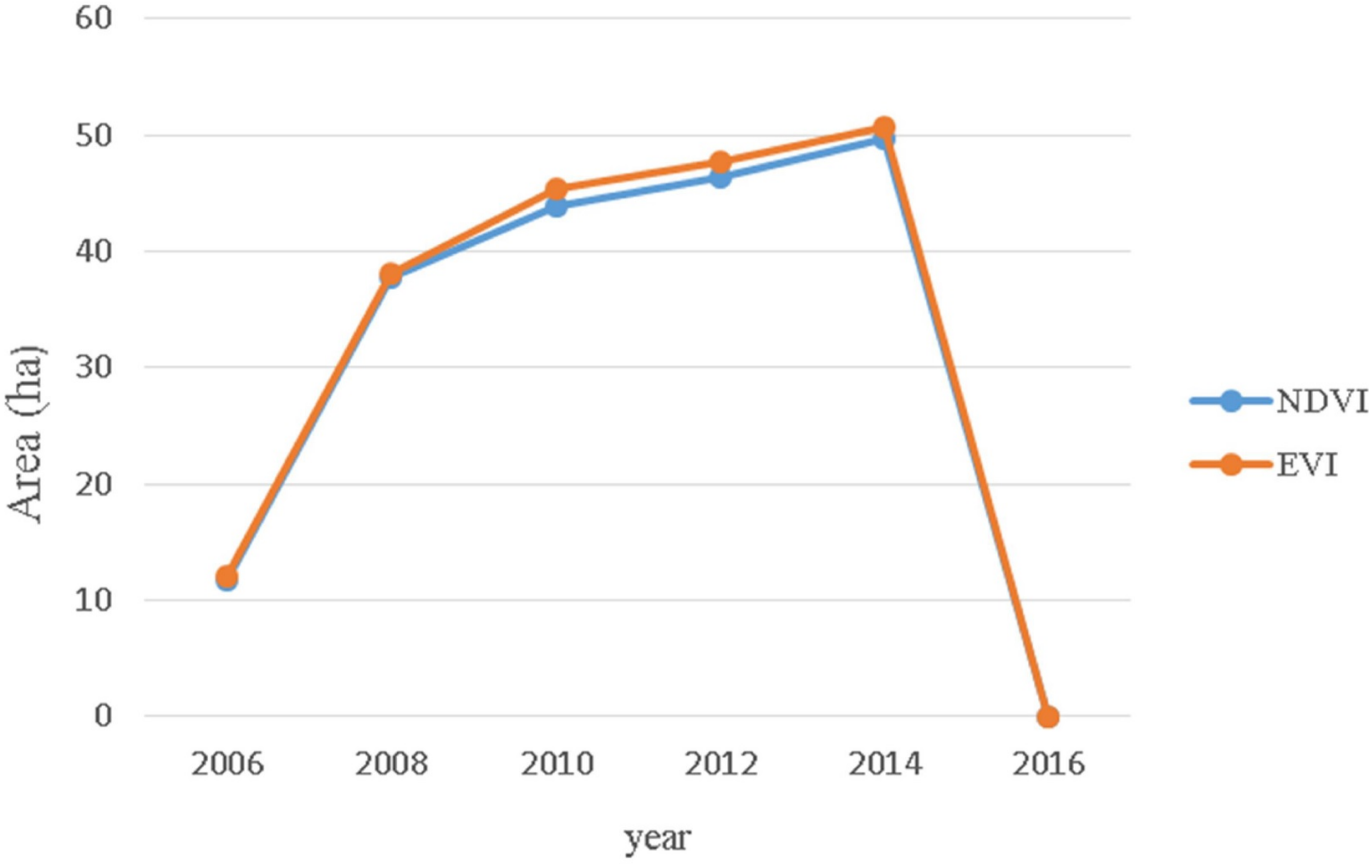
The estimated areas of mangrove by using NDVI and EVI.

Changes of the mangroves over the study decade are shown in Fig. 2. The northern dense region of mangrove appeared in 2006 and then expanded to the south in 2008. In 2010 and 2012, small-scale removal of the estuary of the Sanxing stream can be observed, and the area was estimated at approximately 8 ha In 2014, small-scale removal at the lower end of the dense region was also observed. The other dense region appeared in the south near Haishan Fishing Port in 2008. Sparse seedlings appeared in the area between the two dense regions after 2008. Mangroves were removed manually and thus the mangrove area fluctuated from 2010 to 2014 (Fig. 3). Using NDVI, the areas of mangroves were estimated at 11.7, 49.7, and 0 ha in 2006, 2014, and 2016, respectively (Table 1). The results of EVI are consistent with those of NDVI.

### Biological survey results

**Figure 4:**
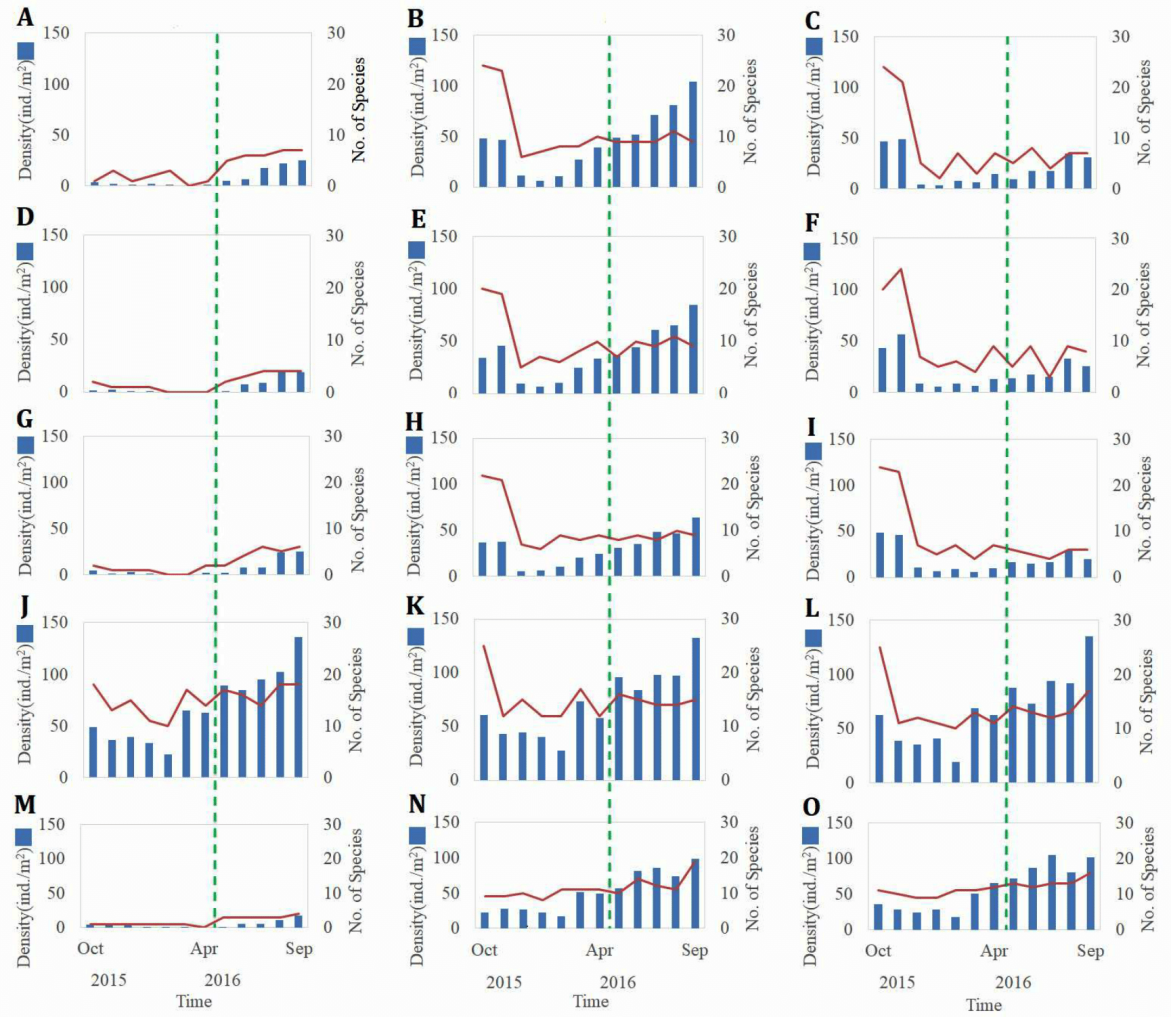
The fluctuations of the monthly density and number of species of marcobenthos. Density and species number of marcobenthos before and after mangrove removal at Site A1 (A); density and species number of marcobenthos before and after mangrove removal at Site A2 (D); density and species number of marcobenthos before and after mangrove removal at Site A3 (G); density and species number of marcobenthos before and after mangrove removal at Site A4 (*J*); density and species number of marcobenthos before and after mangrove removal at Site A5 (M); density and species number of marcobenthos before and after mangrove removal at Site B1 (B); density and species number of marcobenthos before and after mangrove removal at Site B2 (E); density and species number of marcobenthos before and after mangrove removal at Site B3 (H); density and species number of marcobenthos before and after mangrove removal at Site B4 (K); density and species number of marcobenthos before and after mangrove removal at Site B5 (N); density and species number of marcobenthos before and after mangrove removal at Site C1 (C); density and species number of marcobenthos before and after mangrove removal at Site C2 (F); density and species number of marcobenthos before and after mangrove removal at Site C3 (I); density and species number of marcobenthos before and after mangrove removal at Site C4 (L); density and species number of marcobenthos before and after mangrove removal at Site C5 (O).

The fluctuations in monthly benthic density and the number of species are shown in Fig. 4. The benthic density was 1 to 4 ind./m^2^ and the species varied from 0 to 3 at sampling stations A1, A2, A3, and A5 before mangrove removal. The crab species included *Uca arcuata, Uca lacteal*, and *Helice formosensis*. No Bivalvia species were found. After mangrove removal, the benthic density rose to 2 to 25 ind./m^2^ and the species range increased to between two and seven. The increased crab species were *Uca formosensis, Macrophthalmus banzai*, *Mictyris brevidactylus*, and two species of Bivalvia, *Tellina jedonensis* and *Mactra veneriformis* were found*.* These results showed that the benthic density and the number of species increased after mangrove removal. The smallest benthic density occurred in January 2016 and the largest in September 2016. The smallest number of species was found in February 2016 and the largest in August 2016.

In the sampling sites of the non-mangrove region, the benthic density varied from 20 to 60 ind./m^2^, and the species varied from five to 25 before mangrove removal. On the tidal flats in the non-mangrove region, the crab species included *Uca arcuata, Uca lacteal, Mictyris brevidactylus,* and *Macrophthalmus banzai*, and the Bivalvia species included *Laternula anatine, Meretrix lusoria, Cycladicama oblonga, Tellina jedonensis, Mactra veneriformis.* After mangrove removal, the benthic density increased to 25 to 130 ind./m^2^, and the number of species increased to between 10 and 19. Benthic density was smallest in January 2016 and largest in August 2016. The species diversity was largest in October 2015 and smallest in January 2016. These results showed that the benthic density and species change seasonally in Siangshan Wetland, decreasing in winter and spring and increasing in summer.

The non-mangrove region had significantly higher benthic biomass than did the mangrove areas (*t* = 2.45, *p* = 0.003). Likewise, there were significantly fewer species in the mangrove areas than in the non-mangrove region (*t* = 2.75, *p* = 0.04). The benthic biomass was significantly different at periods before and after mangrove removal (one-way ANOVA, *F* = 5.571, *p* = 0.022).

**Figure 5:**
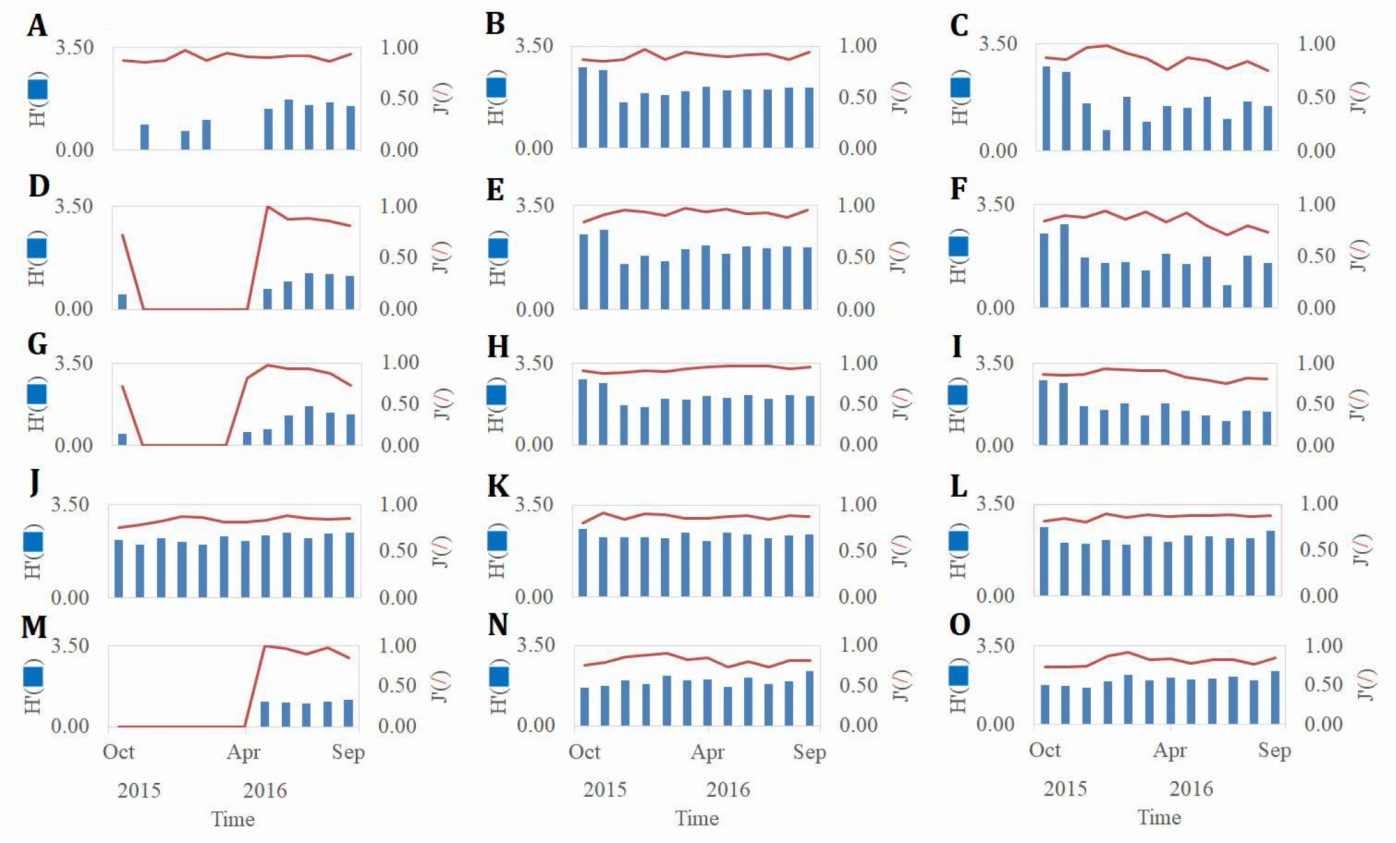
Variations of Shannon–Wiener index (*H*′) and Palou’s evenness index (*J*). Shannon–Wiener index (*H*′) and Palou’s evenness index (*J*) of marcobenthos before and after mangrove removal at Site A1 (A); Shannon–Wiener index (*H*′) and Palou’s evenness index (*J*) of marcobenthos before and after mangrove removal at Site A2 (D); Shannon–Wiener index (*H*′) and Palou’s evenness index (*J*) of marcobenthos before and after mangrove removal at Site A3 (G); Shannon–Wiener index (*H*′) and Palou’s evenness index of marcobenthos before and after mangrove removal at Site A4 (*J*); Shannon–Wiener index (*H*′) and Palou’s evenness index of marcobenthos before and after mangrove removal at Site A5 (M); Shannon–Wiener index (*H*′) and Palou’s evenness index of marcobenthos before and after mangrove removal at Site B1 (B); Shannon–Wiener index (*H*′) and Palou’s evenness index of marcobenthos before and after mangrove removal at Site B2 (E); Shannon–Wiener index (*H*′) and Palou’s evenness index of marcobenthos before and after mangrove removal at Site B3 (H); Shannon–Wiener index (*H*′) and Palou’s evenness index of marcobenthos before and after mangrove removal at Site B4 (K); Shannon–Wiener index (*H*′) and Palou’s evenness index of marcobenthos before and after mangrove removal at Site B5 (N); Shannon–Wiener index (*H*′) and Palou’s evenness index of marcobenthos before and after mangrove removal at Site C1 (C); Shannon–Wiener index (*H*′) and Palou’s evenness index of marcobenthos before and after mangrove removal at Site C2 (F); Shannon–Wiener index (*H*′) and Palou’s evenness index of marcobenthos before and after mangrove removal at Site C3 (I); Shannon–Wiener index (*H*′) and Palou’s evenness index of marcobenthos before and after mangrove removal at Site C4 (L); Shannon–Wiener index (*H*′) and Palou’s evenness index of marcobenthos before and after mangrove removal at Site C5 (O).

The variations in the Shannon–Wiener index (*H*′) and Pielou’s evenness index (*J*) are illustrated in Fig. 5. *H*′ varied from 0 to 1.04, while J varied from 0 to 0.95 before mangrove removal, whereas after mangrove removal, the H’ varied from 0.69 to 1.72, and J varied from 0.77 to 1.00. *H*′ was smallest in January 2016 and largest in June 2016. J was smallest in September 2016 with a value of 0.77. The results showed that both the Shannon–Wiener index (*H*′) and Pielou’s evenness index (*J*) increased, indicating an increase in biodiversity after mangrove removal.

In the non-mangrove region, the Shannon–Wiener index (*H*′) varied from 1.51 to 2.83 and Pielou’s evenness index (*J*) varied from 0.73 to 0.98 before mangrove removal, while after mangrove removal, the Shannon–Wiener index (*H*′) varied from 1.04 to 2.46 and Pielou’s evenness index (*J*) varied from 0.73 to 0.93. These results showed that the Shannon–Wiener index (*H*′) decreased in winter and increased gradually in summer and spring.

**Figure 6:**
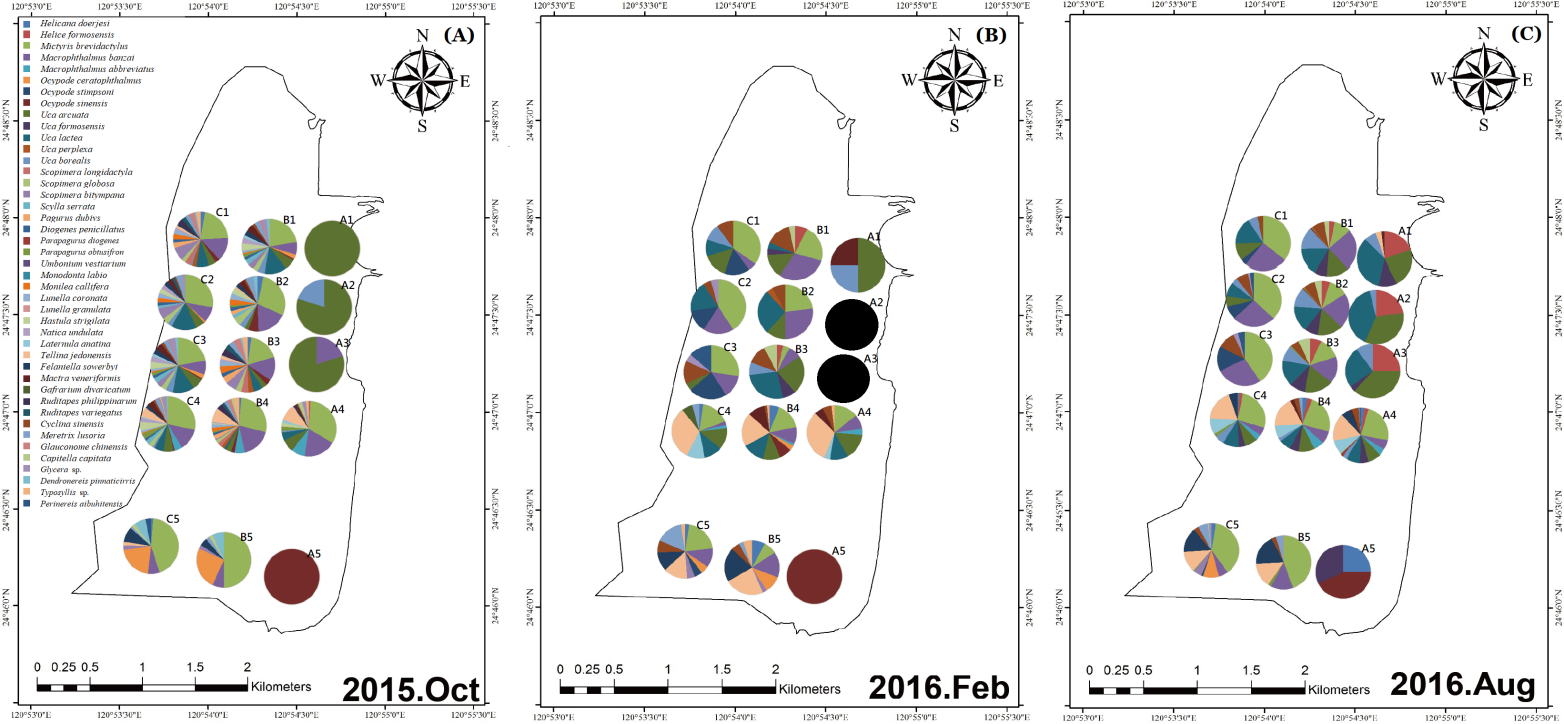
The spatial variation of species and species quantity. The species and species quantity at all sites in October 2015 (A); the species and species quantity at all sites in February 2016 (B); the species and species quantity at all sites in August 2016 (C).

The spatial variations of species composition are shown in Fig. 6. Two crab species, *Uca arcuata* and *Uca vocans borealis*, were observed at the sampling sites within the mangrove area before mangrove removal. After mangrove removal five kinds of crabs were observed, including *Uca arcuata, Uca vocans borealis, Uca formosensis, Uca lactea*, and *Helice formosensis*. Within the non-mangrove regions, species composition varied both spatially and seasonally. The number of species within the mangrove regions increased after mangrove removal.

**Figure 7:**
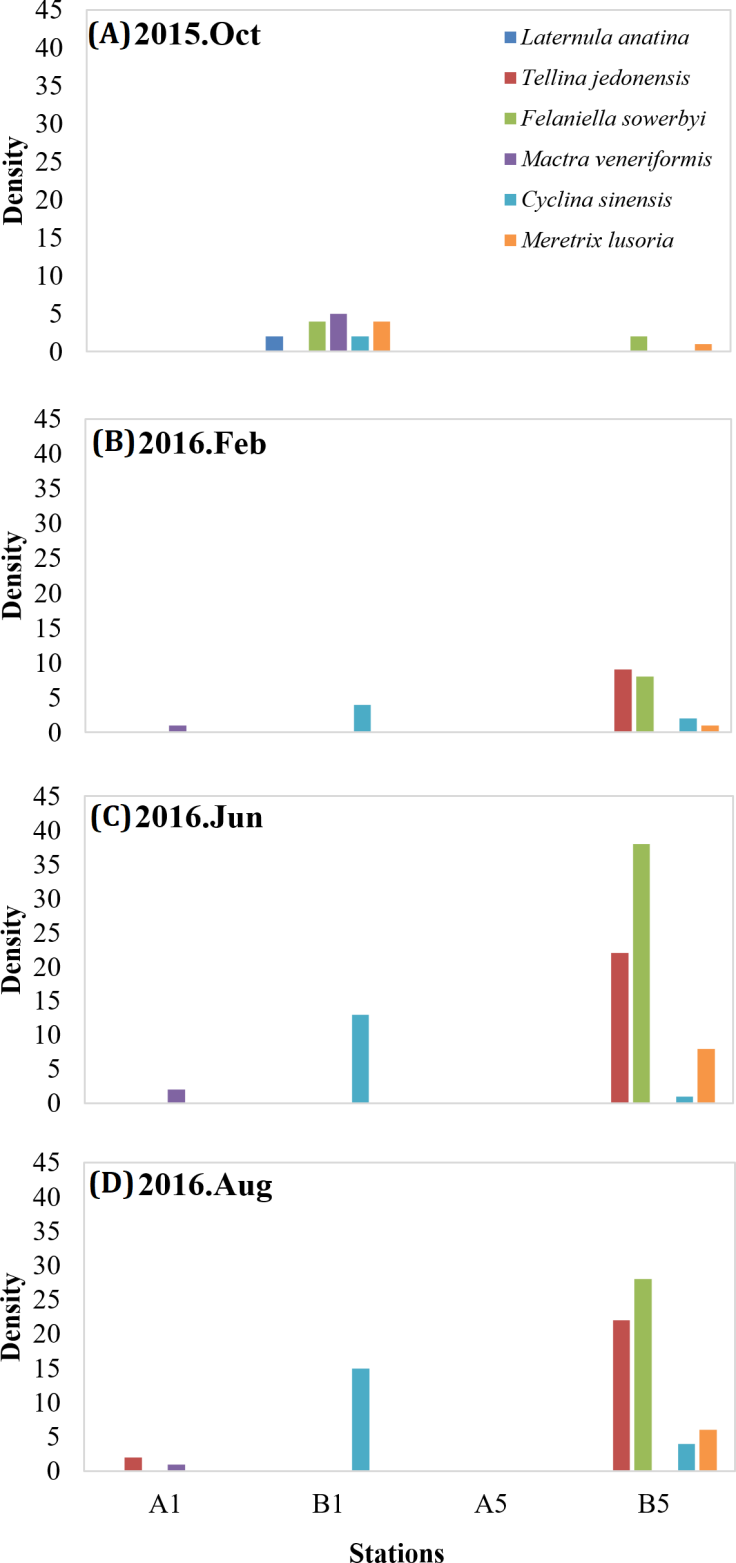
Statistics of bivalve species and the number of species. Species density of bivalve species at A1, B1, A5 and B5 in October 2015 (A); species density of bivalve species at A1, B1, A5 and B5 in February 2016 (B); species density of bivalve species at A1, B1, A5 and B5 in June 2016 (C); species density of bivalve species at A1, B1, A5 and B5 in August 2016 (D).

**Figure 8:**
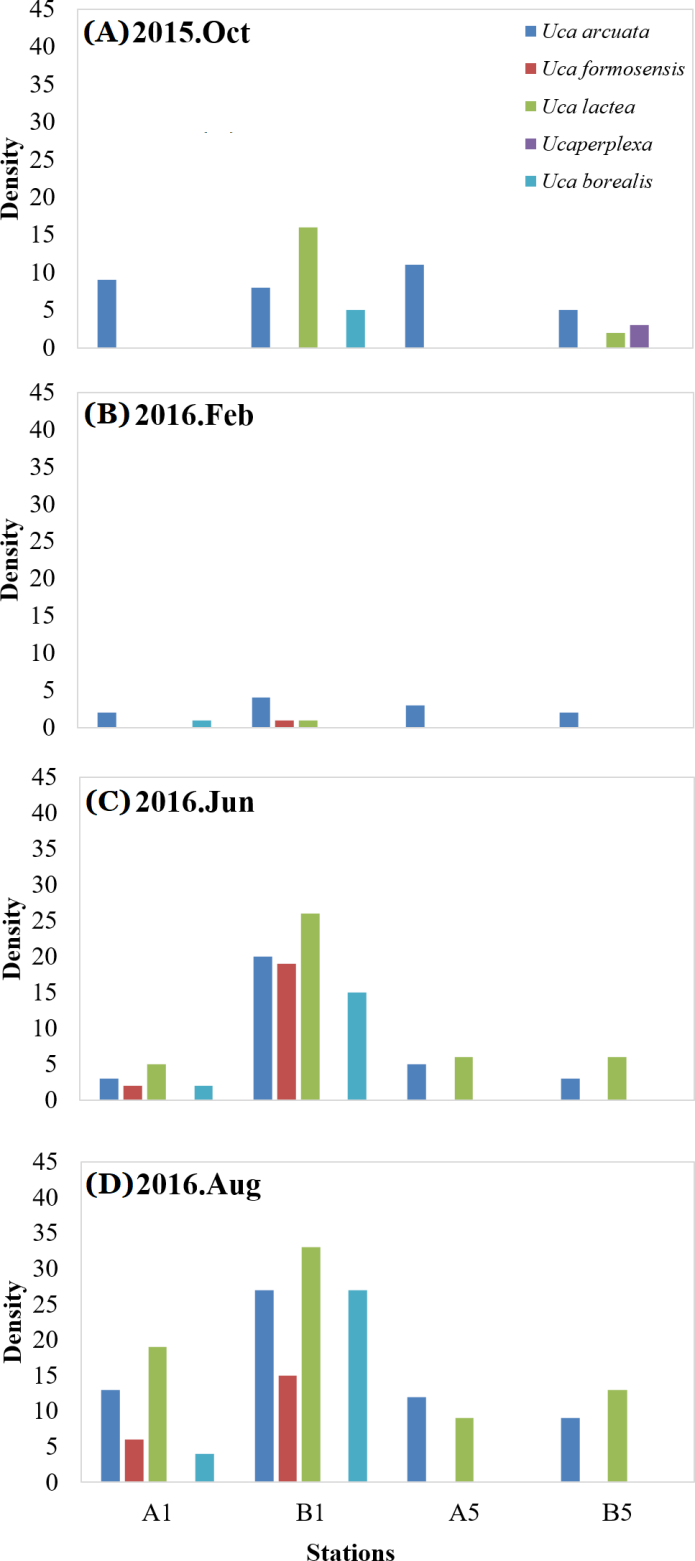
Statistics of tide crabs. Species density of tide crabs at A1, B1, A5 and B5 in October 2015 (A); species density of tide crabs at A1, B1, A5 and B5 in February 2016 (B); species density of tide crabs at A1, B1, A5 and B5 in June 2016 (C); species density of tide crabs at A1, B1, A5 and B5 in August 2016 (D).

Furthermore, the density of bivalve species in the two different regions (mangrove: A1, A5; and non-mangrove: B1, B5) were compared (Fig. 7). Lower density was measured in the mangrove region than in the non-mangrove region. The density of crustacean species varied from month to month (Fig. 8), but the densities measured in the mangrove region were also significantly lower than those in the non-mangrove region. The crabs returned to the original habitat shortly after mangrove removal. These results show that these benthic organisms were forced to migrate from their original habitat to nearby areas due to the spreading of mangroves. After mangrove removal, the species returned to their original habitats.

## Discussion

Satellite telemetry is a technique increasingly being adopted to efficiently observe, quantify, and survey vegetation (Green, Clark & Edwards, 2000; Kovacs, Wang & Blanco-Correa, 2001; Kovacs et al., 2004; Kovacs, Wang & Flores-Verdugo, 2005; Satyanarayana et al., 2011). Subhanil (2016) reported that the NDVI index provided satisfactory results in distinguishing various types of vegetation coverage. Satyanarayana et al. (2011) proved that NDVI and ground-based measurements are a positively related.

In general, mangrove areas have been identified as having high diversity and good ecological value (Ashton & Macintosh, 2002; Macintosh, Ashton & Havanon, 2002) because they provide food and shelter, resulting in rich ecosystems (Duke et al., 2007). The annual market of capture fisheries has been conservatively estimated at between US $750 and $16,750 per ha, which illustrates the potential support value of mangroves (Rönnbäck, 1999). However, some areas have been cut down and their mangroves have been removed to construct aquaculture farms, for coastal development purposes, or by local residents to acquire firewood and other products (Diop, 2003). Furthermore, conservation and restoration works have been undertaken by the mangrove management project to avoid the destruction and degradation of mangrove habitats (Alfaro, 2010). Nevertheless, mangrove removal for the aforementioned purposes clearly differs from the purpose of mangrove removal in this study.

The biological investigation of benthic communities at Siangshan Wetland in 2002 and 2005 showed that the dominant species were *Macrophthalmus banzai, Laternula anatine*, and *Mictyris brevidactylus* (Wilderness Conservation Association, 2007). Compared with prior studies, similar species were found in the non-mangrove region in this study, such as *Uca arcuata, Uca vocans borealis, Uca formosensis, Uca lactea*, and *Helice formosensis*, whose habitats were mainly mudflats and frequently immersed in sea water. An investigation of mangrove removal in 2010 and 2011 showed that benthic habitat gradually changed from mudflats to sandy flats, and biological diversity was significantly higher in in the mangrove-removal area than in the mangrove control area (Young & Zhang, 2014). After mangrove removal, the composition of the sediment changed from a muddy to a sandier habitat, and clams began to appear (Young, 2013).

*Uca arcuate, Uca lacteal*, and *Uca borealis* are common in mangroves, sandy and muddy areas, and salt marshes in Siangshan Wetland. Given their feeding habits, they are crucial for preserving wetland environments: they aerate the substrate and prevent anaerobic stagnation by sifting through the sands (Levinton, Judge & Kurdziel, 1995). In terms of preferred habitat, *Uca arcuate* prefers to appear in a wet environment. This means that it is more likely to appear beside waterways and tidal pools in wetlands after ebb tide. The expansion of mangroves often blocks the waterways. After the mangroves had been removed, clear waterways appeared, and these species naturally returned to their original places.

Similar results were reported for Matapouri Estuary, in the north of New Zealand, where benthic abundance and biodiversity in mangrove habitats had been significantly below those of adjacent seagrass habitats (Alfaro, 2006). The dominant benthic organisms obtain nutrients from various sources, including bacteria and algae; the effect of mangrove-derived nutrients on the food web may be local, with minimal exports of organic material to surrounding habitats (e.g., sandflats) (Alfaro et al., 2006). Alfaro (2010) also reported that mangrove removal in temperate region in the north of New Zealand had particular effects on sediment characteristics and benthic communities. The sediment environment changed almost immediately from muddy to sandy after mangrove removal; other changes included a subsequent overall increase in the number of snails, crabs, and bivalves. Consistent with our results, Alfaro (2010) clearly demonstrated the effects of mangrove removal on the characteristics benthic fauna. The unrestricted spreading of mangroves leads to the reduction and destruction of other habitats and have attracted the attention of many community groups and environmental managers, who considered mangrove expansions to have adverse influences on ecology and socioeconomics (Schwarz, 2003).

Worldwide, mangrove rehabilitation projects are undertaken primarily to revive forest cover and habitat functions (Katon et al., 2000; Barbier, 2006). Rehabilitation and restoration differ in their meaning: the latter refers to partly or completely replacing the structure or functional aspects of an affected ecosystem, whereas the former attempts to return the ecosystem to its original characteristics (Field, 1999). Many rehabilitation projects are conducted by planting full-grown mangroves or seedlings. Rehabilitation projects are most effective when they are appropriate to the environmental conditions (Friess et al., 2012). In this study, the rehabilitation effort of mangrove removal was applied to improve the habitat for benthic organisms.

## Conclusions

The spread of mangroves at Siangshan Wetland in Taiwan is in contrast with the well-documented overall trend of mangrove loss. In the investigated area, the spread of mangroves changed the habitat structure and function for benthic organisms and caused infilling of estuaries, flooding, and small black mosquito breeding. Therefore, the Hsinchu Municipal Government has instituted several small-scale mangrove-removal projects since 2000. A large-scale removal project was undertaken from October 2015 to March 2016. In this study, we used satellite telemetry and biological sampling to monitor the situational differences before and after mangrove removal. The satellite imagery showed that spatial variation occurred during 2006–2016 and the maximum area of mangrove removal was 49.7 ha in 2014. The impacts of mangrove removal on the benthic organisms and adjacent habitats were investigated within Siangshan Wetland between October 2015 and September 2016. The benthic organisms were sampled in habitats in the mangrove and non-mangrove regions both before and after mangrove-removal activities. The non-mangrove region had more number of individuals, number of species, and indicators than did the mangrove region. After mangrove removal, the values for individuals, number of species, and indicators of benthic organisms increased significantly. This study clearly evidences the beneficial effects of mangrove removal on benthic organisms in the Siangshan Wetland in Hsinchu, Taiwan. The results show that mangrove removal can be an appropriate approach for habitat rehabilitation for benthic organisms. The study also provides useful ecological data for coastal managers and other officials interested in controlling mangrove spread.

##  Supplemental Information

10.7717/peerj.5670/supp-1 Supplemental Information 1 Species information data

## Additional Information and Declarations

### Field Study Permissions

The following information was supplied relating to field study approvals (i.e., approving body and any reference numbers): Field experiments were approved by the Environmental Protection Administration, R.O.C. (Taiwan) (Project number: EPA 102-078A).

### Data Availability

The following information was supplied regarding data availability: The raw data are provided in a Supplemental File.
